# Broadband gradient impedance matching using an acoustic metamaterial for ultrasonic transducers

**DOI:** 10.1038/srep42863

**Published:** 2017-02-17

**Authors:** Zheng Li, Dan-Qing Yang, Shi-Lei Liu, Si-Yuan Yu, Ming-Hui Lu, Jie Zhu, Shan-Tao Zhang, Ming-Wei Zhu, Xia-Sheng Guo, Hao-Dong Wu, Xin-Long Wang, Yan-Feng Chen

**Affiliations:** 1 National Laboratory of Solid State Micro-structures & Department of Materials Science and Engineering, Nanjing University, Nanjing 210093, China; 2 Institute of Acoustics, Nanjing University, Nanjing 210093, China; 3 Department of Mechanical Engineering, The Hong Kong Polytechnic University, Hung Hom, Kowloon, Hong Kong SAR, China

## Abstract

High-quality broadband ultrasound transducers yield superior imaging performance in biomedical ultrasonography. However, proper design to perfectly bridge the energy between the active piezoelectric material and the target medium over the operating spectrum is still lacking. Here, we demonstrate a new anisotropic cone-structured acoustic metamaterial matching layer that acts as an inhomogeneous material with gradient acoustic impedance along the ultrasound propagation direction. When sandwiched between the piezoelectric material unit and the target medium, the acoustic metamaterial matching layer provides a broadband window to support extraordinary transmission of ultrasound over a wide frequency range. We fabricated the matching layer by etching the peeled silica optical fibre bundles with hydrofluoric acid solution. The experimental measurement of an ultrasound transducer equipped with this acoustic metamaterial matching layer shows that the corresponding −6 dB bandwidth is able to reach over 100%. This new material fully enables new high-end piezoelectric materials in the construction of high-performance ultrasound transducers and probes, leading to considerably improved resolutions in biomedical ultrasonography and compact harmonic imaging systems.

Ultrasonography, as a non-invasive and non-radiative technology, has opened a new era of real-time diagnostic medical imaging. Currently, it is being applied in broad applications of gynaecologic, intravascular, endoscopic and molecular imaging^1^,^2^,^3^,^4^,^5^,^6^,^7^,^8^,^9^. To generate and receive ultrasound signals, transducers for ultrasonography systems are built with encased piezoelectric materials to convert electrical signals to mechanical vibrations, and vice versa. Typically, the acoustic impedances of piezoelectric materials are one to two orders of magnitude higher than of those of human body tissues. Such an impedance mismatch not only causes the majority of piezo-generated acoustic energy to be reflected at the boundary but also devastatingly lengthens ultrasound pulses and transducer ring-down time^10^,^11^,^12^,^13^.

The single quarter-wavelength matching layer approach has been proposed and extensively studied^14^,^15^,^16^,^17^,^18^ to tackle the problem by taking advantage of destructive and constructive interference of ultrasound. Composite designs that stack two or more layers of quarter-wavelength thick matching materials have also been explored to improve the spectral performance^19^,^20^,^21^,^22^. These quarter-wavelength-matching-based solutions have improved the ultrasound propagation between piezoelectric materials and the target medium. Theoretically, total transmission is achievable at the operating frequency if the matching layer is fabricated with the proper material. However, the transmission coefficient drops off substantially away from the operating frequency, leading to a narrow pass-band window. This has not been an issue for conventional piezoelectric materials whose intrinsic operational bandwidth is confined but definitely poses a threat to the introduction of the new emerging generation of relaxation ferroelectric single-crystal materials such as PMN-PT^23^,^24^,^25^,^26^,^27^,^28^,^29^,^30^,^31^,^32^,^33^,^34^,^35^,^36^. As the best piezoelectric materials to revolutionize medical ultrasound transducers, these single crystals present exceptional piezoelectric performance, providing more than 5 times higher strain energy densities and significantly higher electromechanical couplings than conventional PZT ceramics^27^,^28^. However, with the operational percentage bandwidth of single crystals reaching over 110%, developing a corresponding broadband acoustic impedance matching scheme remains an unsolved matter.

In this paper, we present a type of anisotropic cone-structured acoustic metamaterial as the ideal matching layer for broadband ultrasound transducers. The development and realization of artificial acoustic metamaterials already created many fascinating approaches^29^,^30^,^31^,^32^,^33^. Among them, impedance matching with phononic crystals and acoustic metamaterials has been intensively studied during the last decade to explore the possibility of unity transmittance^34^,^35^,^36^,^37^,^38^. However, the findings are rather limited with respect to the prerequisites such as adjacent mediums and wave incident angles. In addition, most importantly, none of them can provide practical impedance matching solutions for medical ultrasound transducers.

Our anisotropic cone-structured acoustic metamaterial matching layer consists of periodically arranged subwavelength silica-epoxy composite unit cells with the fraction volume ratio of the silica cone decreasing away from the piezoelectric material. It can effectively be treated as an inhomogeneous material whose acoustic impedance gradually changes along the ultrasound propagation direction. This metamaterial matching layer, fabricated by etching the peeled silica optical fibre bundles with hydrofluoric acid solution, is capable of providing a corresponding −6 dB percentage bandwidth as large as 100% by itself. With such a broad passband window, ultrasonography systems can enjoy the full potential of single-crystal piezoelectric materials, especially with regard to better axial resolution and higher mode harmonic imaging.

## Results

### Anisotropic cone-structured acoustic metamaterial matching layer

**Figure 1:**
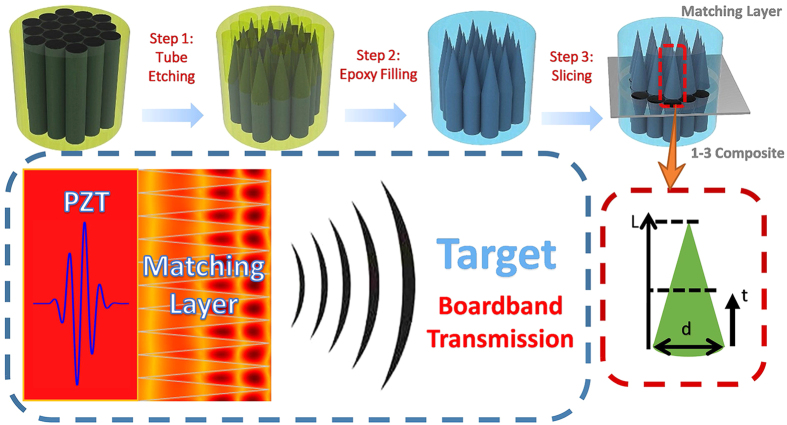
Fabrication of the metamaterial matching layer. The core arrays were first formed using the tube etching method. After filling the voids with epoxy resin (EPO-TEK 301), the metamaterial matching layer was then extracted via precise cutting and integrated with a piezoelectric material unit to test the broadband acoustic transmission performance. The structure was modelled as a pyramidal cone with a diameter d of 125 μm and taper length L = 610 μm.

The unit cell of the cone-structured acoustic metamaterial is formed by a pyramidal cone structure; its structural model is shown in Fig. 1, and its geometric properties can be described by an overall length *L* and a base diameter *d* that is sufficiently smaller than the operating acoustic wavelength. From the effective medium approach, this anisotropic metamaterial can be treated as having a gradient change of equivalent acoustic impedance. To arrange the unit cells in a periodic fashion and make the acoustic metamaterial matching layer suitable for biomedical ultrasonography applications, the base diameter *d* has to be on the deep sub-millimetre scale due to the high operating frequencies of the transducers. Therefore, we first etched a bunch of cylindrical silica fibres into two-dimensional arrays of pyramidal cones using a tube array fabrication and etching method^39^. The formation mechanism of these pyramidal cones stems from the microfluidic behaviours of the etchants. By adjusting the concentration of the etching liquid, different gradient profiles of the pyramidal cones could be formed^40^. Later, the space between the pyramidal cones was filled with epoxy resin via a vacuum treatment. With precision cutting and polishing, the cone-structured acoustic metamaterial matching layer was finally extracted, as shown in Fig. 1.

**Figure 2:**
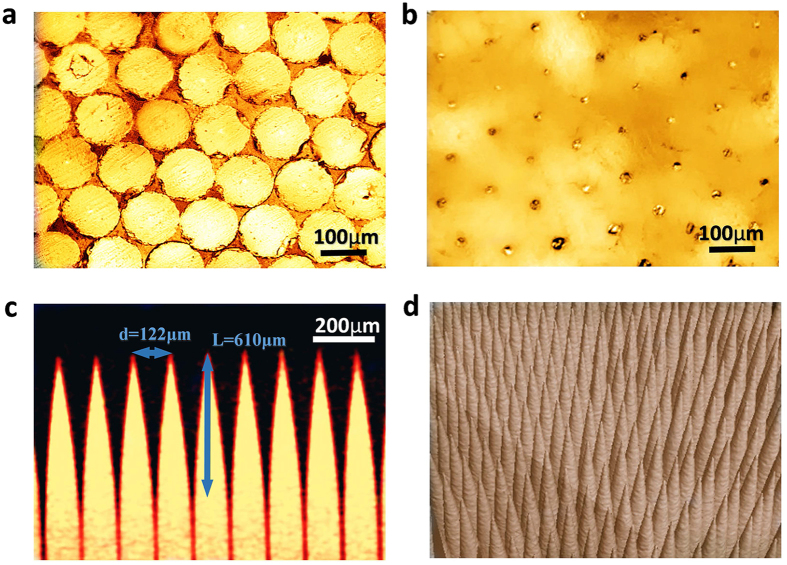
Structural characterization of the pyramidal cone metamaterial matching layer. Under the optical microscope, the bottom side of the matching layer (**a**) is hexagonal close-packed silica columns. This side of the matching layer has the maximum acoustic impedance, which is approximately equal to that of silica. (**b**) On the top side of the matching layer, barely visible tiny dots, which represent the tips of the silica fibres, were regularly spread in the epoxy resin matrix. This side has the lowest acoustic impedance, which is equal to that of epoxy resin. (**c**) The cross-sectional image of the metamaterial matching layer was acquired via an industrial CT test. The pyramidal cone array has a cycle length of 122 μm, and the tip length of the pyramidal cone is 610 μm. (**d**) The 3-D topography of the pyramidal cone arrays embedded in the matching layer.

The fabricated metamaterial matching layer was investigated by optical microscopy and computed tomography tests. From the images of parallel cross-sectional surfaces shown in Fig. 2a and b, different morphologies of silica embedded in the epoxy resin matrix can be clearly observed on the two surfaces. On the bottom side bordering the piezoelectric material, the tightly packed hexagonal silica fibre cores occupy over 90% of the total area. The equivalent specific acoustic impedance reaches the maximum value^41^. In contrast, on the other side adjacent to the target medium, the tips of the silica fibre cones are barely visible as small dots regularly spread in the epoxy resin matrix; thus, the properties of the epoxy resin dominate the lower effective acoustic impedance there. A vertical cross-section image and the 3-D topography of the cone-structured acoustic metamaterial matching layer are shown in Fig. 2c and d. One can easily observe the well-crafted three-dimensional geometry and topology of the pyramidal silica fibre cone unit cell array. We measured the lengths *L* of the silica fibre pyramidal cones to be approximately 610 μm and the period of the cone arrays to be approximately 122 μm, in agreement with the diameter *d* of the silica fibre cone base. The shape of the silica fibre cones and the uniformity of the distribution are both well controlled by the proposed fabrication technique. As a result, the effective acoustic impedance of this metamaterial matching layer can be regarded as decreasing gradually, from high impedance near the silica-dominated bottom side to low impedance close to the epoxy-dominated top side.

### Gradient impedance

To theoretically calculate the equivalent acoustic parameters, the cone-structured acoustic metamaterial matching layer was treated as a 1–3 composite with a varying component ratio. FEM simulation by COMSOL Multiphysics was performed to calculate the equivalent acoustic parameters of a 1–3 composite with different component filling ratios (please refer to the Supplementary Figures S1, S2 and S3), and the results agree well with the ISO-STRAIN theory. Therefore, the specific acoustic impedance *Z* and the longitudinal velocity *V* of a 1–3 composite with different component filling ratios can be approximately obtained from the following equations^42^:


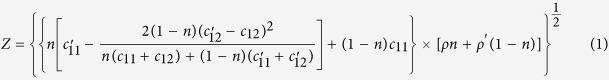



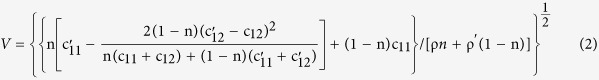


Here, *C*_*ij*_ and 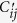 (*i, j* = 1, 2) are the elasticity coefficients of the two components that constitute the metamaterial, *ρ* and *ρ*′ denote the densities, *n* is the volume fraction of one component (silica in our case), and 1 − *n* is that of the epoxy resin.

For our metamaterial matching layer, the volume fraction of silica *n* decreases with the distance *t* from the bottom side of this matching layer. Their relationship with cone length *L* is:


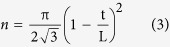


**Figure 3:**
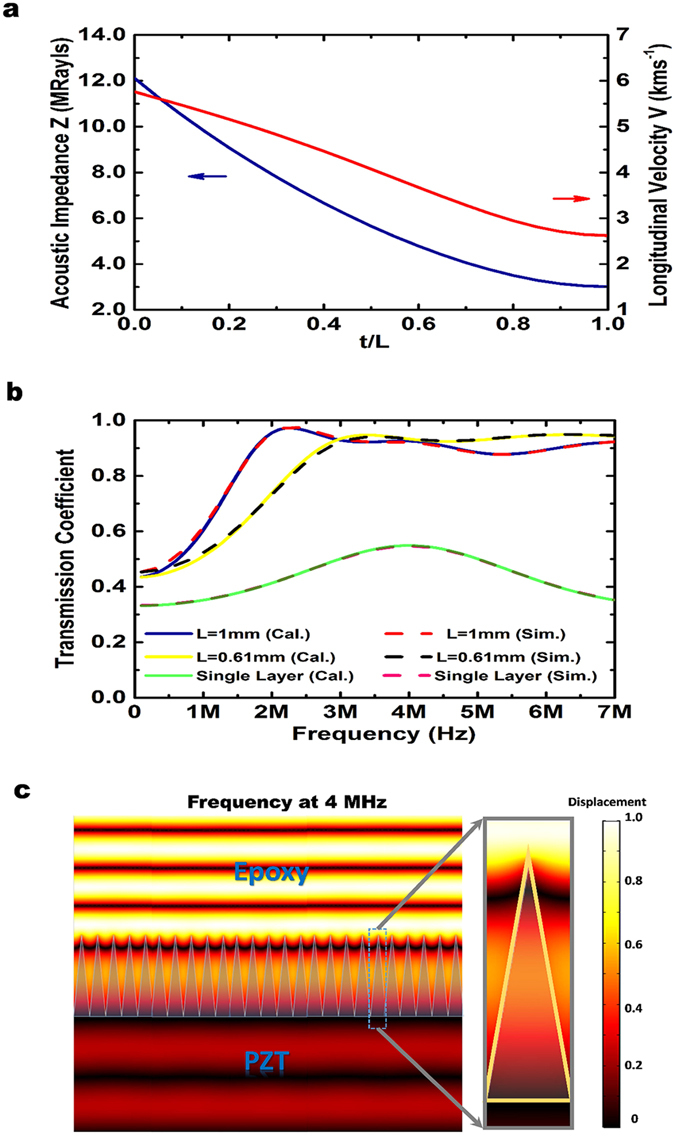
Characteristics of the pyramidal cone metamaterial matching layer. (**a**) Distribution of the equivalent acoustic parameters along the thickness direction. Both the specific acoustic impedance and the longitudinal velocity vary continuously and monotonically. (**b**) Sound transmittance through this metamaterial matching layer. The simulated and theoretical results are indicated by dashed and solid lines, respectively. The transmission performance of the matching layers with thicknesses of 610 μm (solid yellow and dashed black) and 1000 μm (solid blue and dashed red) show better broadband transmissions than the single matching layer (solid green and dashed pink) (**c**) The distribution of the displacement field in this matching layer. Plane incident acoustic waves remain intact after travelling through the metamaterial matching layer, as the high diffraction orders of the scattering waves are essentially confined to the surfaces of the cone structures.

Substituting the material mechanical parameters of silica (Density *ρ* = 2200 kgm^−3^; Young’s modulus *E* = 72 GPa; Poisson’s ratio *σ* = 0.17) and epoxy resin (ρ = 1150 kgm^−3^; *E* = 4.7 GPa; *σ* = 0.36)^43^, the equivalent acoustic impedance and longitudinal velocity change of the metamaterial matching layer along the wave propagation direction can be calculated. Both the equivalent acoustic impedance and the longitudinal velocity presented in Fig. 3a decrease continuously and monotonously with *t*, demonstrating a gradient change of the effective medium whose acoustic properties change from epoxy resin-like to silica-like.

### Transmission through acoustic metamaterial matching layer

The transmission coefficient of an acoustic wave through this gradient impedance acoustic metamaterial matching layer can be expressed by:


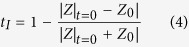


Here, *Z*|_*t*=0_ represents the acoustic impedance of the matching layer at its interface with the incident material, and Z_0_ represents the impedance of the incident material. Because the acoustic metamaterial matching layer is anisotropic in terms of the bulk modulus and density along the wave propagation direction, we obtain the impedance *Z*|_*t*=0_ by satisfying an impedance transfer equation (please refer to the Supplementary Note 2):


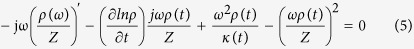


Here, *Z*|_*t*=0_ is the acoustic impedance of the incident boundary, which can be calculated based on the boundary condition that the acoustic impedance of the exit boundary equals the material acoustic impedance of the exit material. To simplify the problem, if we only considered the longitudinal mode and by analogy with compressional waves in a fluid, the bulk modulus κ(t) can be replaced by the equivalent elastic matrix coefficient 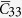 of the 1–3 composite mentioned in the previous section (please refer to the Supplementary Note 2).


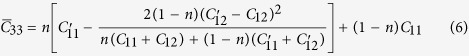


We calculated the spectral transmission performance of the metamaterial matching layer with the theoretical impedance transfer equation model using Mathematica. Full wave numerical simulations using COMSOL Multiphysics were also performed to verify the theoretical model. It is assumed that the matching layer is sandwiched between the piezoelectric ceramic (*ρ* = 6300 kg m^−3^; *E* = 38.4 GPa; *σ* = 0.36) and the epoxy resin. The two resultant transmittance curves of the matching layers with thicknesses of 610 μm and 1000 μm presented in Fig. 3b show similar variations with the operating acoustic wave frequency. For the cone-structured acoustic metamaterial matching layer with a thickness of 610 μm, consistent high transmission over a wide frequency range can be obtained after the first resonance peak at approximately 3 MHz, effectively eliminating the sharp dips in the traditional quarter-wavelength matching condition. However, for the matching layer with a thickness of 1000 μm, an additional 390 μm thick 1–3 composite layer was retained before the impedance gradient layer. The first resonance peak dropped down to approximately 2 MHz with a much higher transmission coefficient near 100%, and after that, high transmissions of approximately 90% are maintained extending into the higher frequency range. Therefore, choosing a matching layer with a thickness of 1000 μm and a piezoelectric ceramic slab with a centre frequency of, e.g., 4 MHz, is sufficient. By coupling these two resonance peaks, wider frequency bandwidth transducers can be achieved.

Moreover, due to the periodic structure of the cone arrays, the metamaterial matching layer will not change the wave front shape and direction of the incident beam. As demonstrated by the full wave simulation results presented in Fig. 3c, plane incident acoustic waves remain intact after travelling through the metamaterial matching layer. The high diffraction order components are essentially confined to the cone surface of the matching layer and eventually become evanescent waves^44^.

### Transducer equipped with acoustic metamaterial matching layer

**Figure 4:**
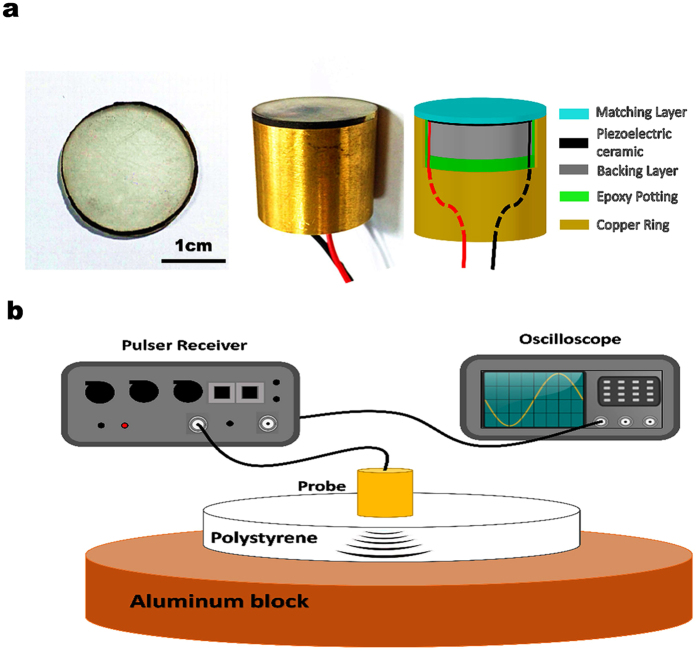
Transducer sample and performance test. (**a**) The fabricated matching layer has a diameter of 20 mm and a thickness of 1000 μm. A transducer was fabricated to demonstrate the performance and practical use of this new metamaterial matching layer. A low acoustic impedance porous piezoelectric ceramic slab with a centre frequency of 4 MHz was used in this transducer. The acoustic impedance of the backing material is 7.8 MRayls. (**b**) The pulse echo tests were conducted on a 1 cm thick polystyrene test block. An aluminium plate acted as the acoustic total reflection body. The traditional λ/4 acoustic impedance matching layer transducer was tested using the same equipment for comparison.

**Figure 5:**
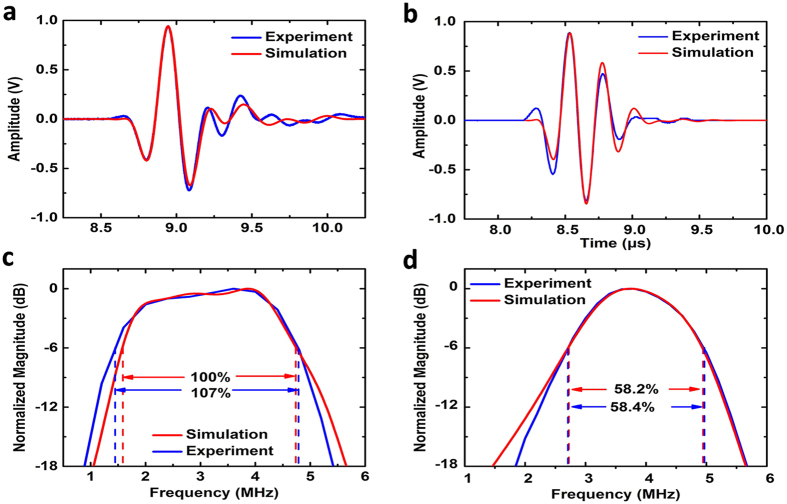
Experimentally measured and simulated results. (**a**,**b**) The echo pulse of the metamaterial matching layer transducer and the single λ/4 matching layer transducer. The measured and simulated results agreed with each other very well. (**c**,**d**) The frequency responses of the transducers on the polystyrene test block. This metamaterial matching layer transducer has a measured −6 dB bandwidth of 107% and simulated bandwidth of 100%. Under the same conditions, the −6 dB bandwidth of the traditional λ/4 matching layer transducer was 58.4% in the experiment and 58.2% in the simulation.

To demonstrate the capability of the cone-structured acoustic metamaterial gradient impedance matching layer, we assembled two 4 MHz piezoelectric ultrasound transducers. As the maximum acoustic impedance of the metamaterial matching layer is much lower than that of traditional piezoelectric ceramics (30–38 MRayls), a low density porous lead zirconate titanate piezoelectric ceramic (PZT) was selected to reduce the impedance mismatch between the piezoelectric material and the matching layer. This piezoelectric material has a relatively low acoustic impedance of approximately 20.0 MRayls (with a density of 6300 kgm^−3^ and a longitudinal velocity of 3200 ms^−1^). A backing layer with an acoustic impedance of 7.8 MRayls was used to eliminate the influence of backward propagating waves. The structure of the transducer is shown in Fig. 4a. One of the transducers was equipped with the cone-structured acoustic metamaterial matching layer, and one side of the acoustic impedance was experimentally measured to be 11.4 MRayls (please refer to the Supplementary Note 1) when the volume fraction of silica *n* was 0.906, which is close to the theoretically calculated 12.2 MRayls; the impedance of the other side was measured to be 3.0 MRayls. Another transducer was assembled with the same piezoelectric material and backing units but a traditional quarter-wavelength matching layer. We tested both transducers using the pulse-echo method with the setup presented in Fig. 4b^45^. Polystyrene was chosen as the external target medium because it is a common material used to manufacture wedge blocks in ultrasonic non-destructive tests and it has a relatively low acoustic impedance and acoustic attenuation. By generating and receiving the pulse signal with the same transducer, we were able to acquire the intrinsic two-way ultrasound transmission performance. The received pulse signals by both transducers are shown in Fig. 5. The transmission coefficients over the spectrum were later obtained using FFT. From the experimental results, the performance differences between the two transducers are obvious. When we test the transducer with one traditional quarter-wavelength single matching layer, the signal received is strong close to the operating frequency of 4 MHz. However, the signal decreases substantially away from the transmission peak with the two-way −6 dB percentage bandwidth measured to be lower than 60%. In contrast, the transducer equipped with the cone-structured acoustic metamaterial matching layer is able to show strong and consistent signals between 1.5 MHz and 5 MHz. The two-way −6 dB percentage bandwidth is measured to be much wider at approximately 107%. Meanwhile, simulations of the transducers’ pulse echo tests were carried out using Piezocad Pro.4.01. The gradient metamaterial matching layer was discretized into 10 sublayers, and the equivalent acoustic parameters and thickness of each sublayer are shown in Table 1. The simulated −6 dB bandwidth of the metamaterial matching layer transducer and the single λ/4 acoustic impedance transducer are 100% and 57.2%, respectively. These results are in good agreement with the measured results. Considering the fact that all of the components of the two transducers, except the matching layers, are identical, it can be concluded that a large portion of the improvement in the spectral performance of the transducer is due to the introduction of our cone-structured acoustic metamaterial matching layer.

## Discussion

In this work, we have designed and fabricated an anisotropic matching layer with a cone-structured acoustic metamaterial for emerging broadband high-performance ultrasound transducers. The model of a metamaterial matching layer was thoroughly investigated through rigorous theoretical analysis, full wave numerical simulation and experimental verification. We also developed an impedance transfer equation to predict acoustic wave transmission through this type of gradient impedance material. This kind of gradient impedance matching layer using an acoustic metamaterial can be built by etching silica fibres into pyramidal cones and then filling the voids with epoxy resin, resulting in a continuous equivalent acoustic impedance change from 11.4 MRayls to 3.0 MRayls across its thickness direction. Further optimization can be made by changing the raw materials for different maximum and minimum acoustic impedances. For example, silicon rubber might be used to replace the epoxy resin for underwater applications because of it has similar acoustic characteristics to those of water; alternatively, silicon might be used instead of silica fibres to improve the maximum equivalent acoustic impedance for PMN-PT single-crystal transducers.

When attached to a piezoelectric material unit, by coupling its broadband transmission peak to the resonance peak of the piezoelectric material, this acoustic metamaterial matching layer shows the ability to provide a high transmission window for through acoustic waves over a broad frequency range. It is experimentally proved that a transducer equipped with this acoustic metamaterial matching layer is able to provide much better spectral performance than the traditional quarter-wavelength techniques, eliminating the major bottleneck in broadband ultrasound transducer design. Meanwhile, simulation of the PMN-PT single-crystal transducers equipped with a silicon epoxy gradient metamaterial matching layer was also conducting by Piezocad using the parameters shown in Supplementary Table S1. Because of the high electromechanical coupling coefficient, its simulated −6 dB bandwidth is 116% (please refer to Supplementary Figure S4). This value is much higher than that of the transducers with single traditional λ/4 impedance matching layer (58%) and is also much better than the experimentally measured −6 dB bandwidth of a double traditional λ/4 impedance matching transducers (77%)^46^. Our work provides a promising matching layer solution based on metamaterials for next generation single-crystal piezoelectric ultrasound transducers.

## Methods

### Fabrication of the acoustic metamaterial matching layer

Silica fibres with diameters of 125 μm were cut into pieces and immersed in acetone to swell the organic coatings. Then, they were bunched to form a hexagonal close-packed structure. Subsequently, the space between these fibre cores was filled with rosin at a temperature of 180 °C for 2 hours with vacuum assistance. The solid rosin-filled fibre bundles were cut into slices and etched in hydrofluoric acid solution for 2.5 hours. After removing the rosin in ethanol for 24 hours, epoxy was poured on the surface of the silica cone array. Vacuum treatment was applied to make sure the space between fibres was filled with epoxy resin (EPO-TEK 301). After the epoxy was cured, the transition structure was extracted using a precision cutting process. By polishing to the proper thickness, a metamaterial acoustic matching layer was formed.

### Structural characterization

Optical microscope images were collected using a metallomicroscope UOP (model UB200i, UOP Photoelectric Technology, Chong Qing, China), and the computed tomography tests were carried out using a Diondo (model d2, Diondo Limited Company, Germany).

### Numerical calculation and FEM simulations

By substituting the effective elastic parameters 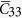 solved by equation 6, the interface impedance Z between the piezoelectric ceramic and the matching layer can be conveniently obtained from by the impedance transfer equation using Mathematica, and then, the calculated transmittance is derived from equation 4.

Three-dimensional simulations of acoustic wave propagation through the matching layer were carried out by FEM software COMSOL Multiphysics. The full wave simulation is based on the same structure of our fabricated sample and the same material parameters.

### Transducer fabrication, transducer simulation and experimental setup

A piezoelectric ceramic slab with the centre frequency of 4 MHz is sandwiched between the heavy acoustic backing and a metamaterial matching layer. The acoustic backing has an acoustic impedance of 7.8 MRayls, and the total thickness of this metamaterial matching layer is 1 mm. An additional 390 μm composite layer was added before the transition layer to further reduce the cutting off frequency of the matching layer. The whole system was then housed in a copper ring by encapsulating epoxy material.

The performance of this transducer was characterized using the pulse-echo method. An aluminium plate (thickness 2 cm) was used as the acoustic total reflection body. The transducer was excited by the ultrasonic pulser receiver (Olympus Panametrics 5073PR), and the received echo was displayed in the oscilloscope (Tektronix TDS2022C). The data were then transferred to the computer and analysed. The measurements were carried out on a 1 cm thick polystyrene test block.

For comparison, a piezoelectric transducer with the same piezoelectric unit, backing and a traditional λ/4 acoustic impedance matching layer (4.2 MRayls) was also fabricated with the same procedure. Its performance was characterized using the same method.

The simulation of the transducer’s pulse echo test was performed using Piezocad Pro.4.0.1 by dividing the gradient matching layer into 10 sublayers. Each sublayer has homogeneous acoustic properties, and the equivalent materials’ parameters listed in Table 1.

## Additional Information

**How to cite this article:** Li, Z. *et al*. Broadband gradient impedance matching using an acoustic metamaterial for ultrasonic transducers. *Sci. Rep.* **7**, 42863; doi: 10.1038/srep42863 (2017).

**Publisher's note:** Springer Nature remains neutral with regard to jurisdictional claims in published maps and institutional affiliations.

## Supplementary Material

Supplementary Information

## Figures and Tables

**Table 1:** Simulation parameters of the gradient metamaterial matching layer transducer.

Layer	ρ (kg/m)	V(m/s)	Z (MRayls)	t (mm)
Piezoelectric Ceramics	6300	3200	20.16	0.395
Matching Layer 1	2102	5763	12.11	0.39
Matching Layer 2	1921	5435	10.44	0.0675
Matching Layer 3	1759	5089	8.95	0.0675
Matching Layer 4	1617	4708	7.61	0.0675
Matching Layer 5	1493	4293	6.41	0.0675
Matching Layer 6	1388	3853	5.35	0.0675
Matching Layer 7	1302	3413	4.44	0.0675
Matching Layer 8	1236	3016	3.73	0.0675
Matching Layer 9	1188	2728	3.24	0.0675
Matching Layer 10	1150	2621	3.01	0.0675
Backing Layer	3900	2000	7.8	20

Simulation of the gradient metamaterial matching layer transducer was carried out through Piezocad by discretizing the matching layer into 10 sub-layers, and each sub-layer could be regarded as a homogeneous material with the acoustic parameters presented in this table.
