# Supplementary material for: Broadband gradient impedance matching using an acoustic metamaterial for ultrasonic transducers
Source: Sci Rep. 2017 Feb 17;7:42863. doi: 10.1038/srep42863 (PMC5314352; doi:10.1038/srep42863)
Supplement: Supplementary Information [file srep42863-s1.pdf]

Supplement information for

Broadband gradient impedance matching using an acoustic metamaterial for ultrasonic transducers

Zheng Li[1], Dan-Qing Yang[2], Shi-Lei Liu[2], Si-Yuan Yu[1], Ming-Hui Lu[1,*], Jie Zhu[3,*], Shan-Tao Zhang[1], Ming-Wei Zhu[1], Xia-Sheng Guo[2], Hao-Dong Wu[2], Xin-Long Wang[2], Yan-Feng Chen[1]

[1] *National Laboratory of Solid State Micro-structures & Department of Materials Science and Engineering, Nanjing University, Nanjing 210093, China*

[2] *Institute of acoustics, Nanjing University, Nanjing 210093, China*

[3] *Department of Mechanical Engineering, The Hong Kong Polytechnic University, Hung Hom, Kowloon, Hong Kong SAR, China*

Correspondence and requests for materials should be addressed to M.-H.L. (email: luminghui@nju.edu.cn) or to J.Z. (email: jiezhu@polyu.edu.hk)

Supplementary Figures

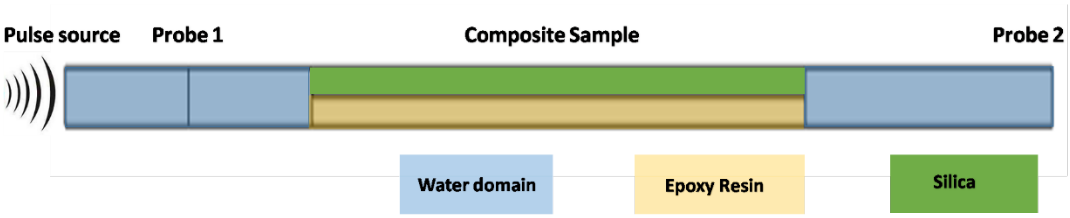

Supplementary Figure S1.Simulation model to calculate the equivalent acoustic parameters of the 1-3 composite. The equivalent acoustic parametersof 1-3 composite with different component filling ratio was carried out by FEM simulation with COMSOL Multiphysics. A guass shape wave with the center frequency 5 MHz was used as the test pulse, and composite sample (a unit cell with a periodic width of 125 μm and a much larger thickness than the wavelength) was inserted at the center of the water domain, two probes was located at the front and back side of the sample to detect the incident (S), reflect (R) and the transmit (T_1&T_2) pulse respectivelywhich are showed in Supplementary Figure S2.

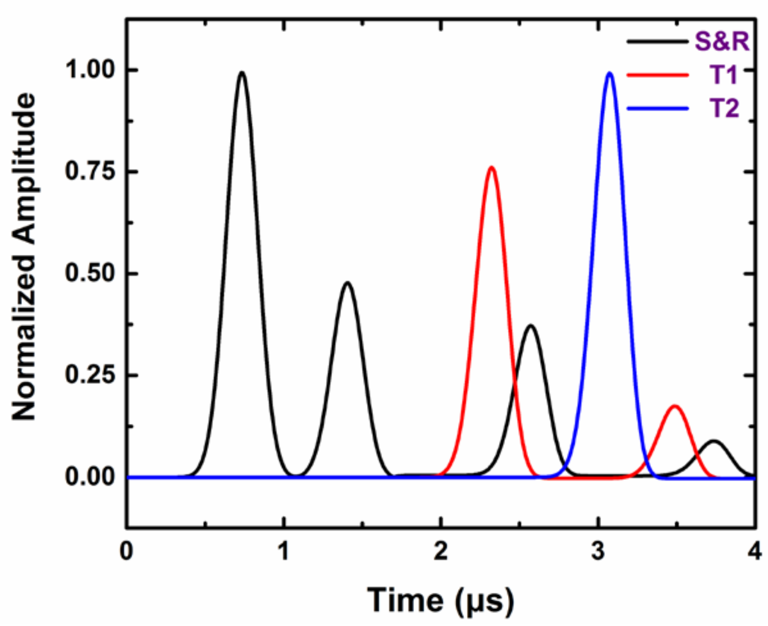

Supplementary Figure S2. The received incident (S), reflect (R) and the transmitted (T_1&T_2) pulse by the acoustic probe in front and at the back side of the sample composite respectively. Here, S means the incident pulse and R represent the reflected pulse from the composite of probe 1, T_1 and T_2 means the received pulse of probe 2 before and after inserting the sample composite.

Equivalent longitudinal velocity c_l of the compositecan be calculated by formula $c_l = \frac{c_w \cdot l}{c_w \cdot \Delta t + l}$ here, $c_w$is the sound velocity of water (1500ms^{-1}) and l is the thickness of the sample which is much larger than the wavelength. Δt is the time difference of the pulse T_1 and T_2. Meanwhile, the equivalent acoustic impedance can be obtained by formula $Z = \frac{1+r}{1-r} Z_{water}$ here,$Z_{water}$is the acoustic impedance of water (1.5MRayls). And r is the reflection coefficient of the composite that can be defined as the contrast of the amplitude of reflect wave A_R and the incident wave A_S $r = \frac{A_R}{A_S}$.

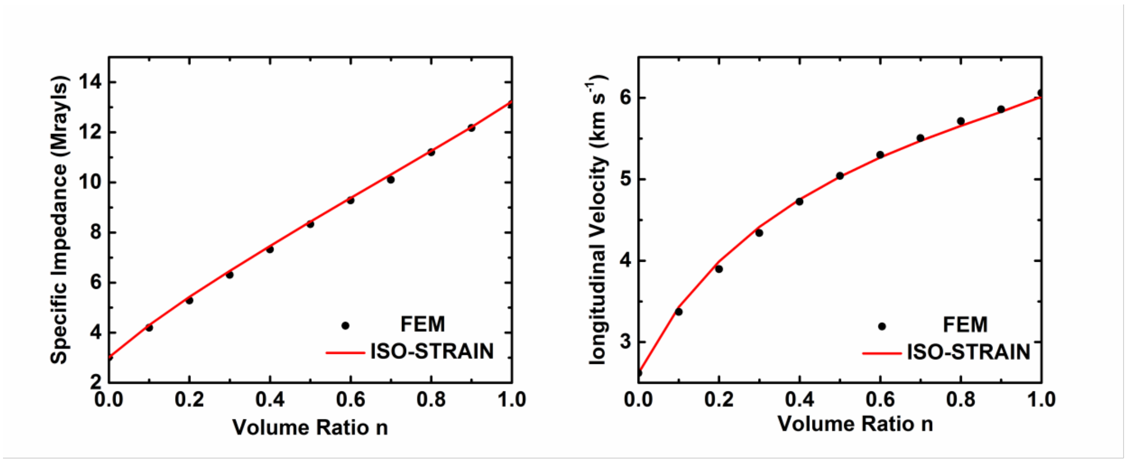

Supplementary Figure S3. Simulated results of the equivalent acoustic parameters of the 1-3 composite. This simulated result of the composite equivalent parameters shows a good agreement with the ISO-STRAIN theory[1]. And as an equivalent homogeneous medium approximation, its specific acoustic impedance *Z* and the longitudinal velocity *V*can be expressed by the formula:

$$Z = \left\{ \left\{ n[c_{11}^{'} - \frac{2(1-n)(c_{12}^{'} - c_{12})^2}{n(c_{11}+c_{12}) + (1-n)(c_{11}^{'}+c_{12}^{'})}] + (1-n)c_{11} \right\} \times [\rho \cdot n + \rho^{'}(1-n)] \right\}^{\frac{1}{2}}$$ and

$$V = \left\{ \left\{ n[c_{11}^{'} - \frac{2(1-n)(c_{12}^{'} - c_{12})^2}{n(c_{11}+c_{12}) + (1-n)(c_{11}^{'}+c_{12}^{'})}] + (1-n)c_{11} \right\} / [\rho \cdot n + \rho^{'}(1-n)] \right\}^{\frac{1}{2}}$$

Here C_{ij} and C_{ij}’ (*i*,*j* = 1, 2) are respectively the elasticity coefficients of silica and epoxy resin that constitute the metamaterial. *ρ* and *ρ’* denote their densities, *n* is the volume fraction of silica, and 1-*n* is that of the epoxy resin.

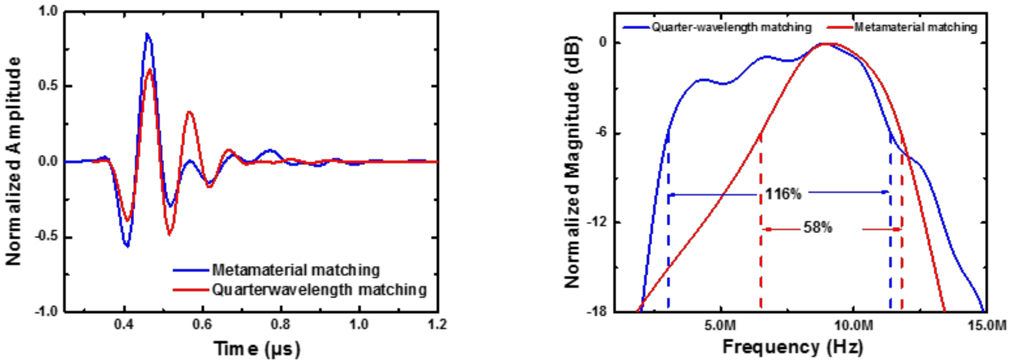

Supplementary Figure S4. Simulated pulse echo results of the PMN-PT single crystal transducer with the silicon epoxy metamaterial gradient matching layer and the traditional single λ/4 matching layer transducer is present for comparison. because of the high electromechanical coupling coefficient of PMN-PT single crystal, simulated results of the gradient metamaterial matching layer transducer has a much shorter pulse width and its -6dB bandwidth in the frequency spectrum is 116%. While the bandwidth of a compared traditional single λ/4 matching layer transducer is only 58% with a much longer pulse width. This simulation results show the potential application value of the gradient metamaterial matching layer for high end piezoelectric ultrasonic transducers

Supplementary Table

Layer	ρ (kg m^{-1})	V(m s^{-1})	Z (MRayls)	t (mm)	K_{33}	ε_r
PMN-PT	8100	4000	32	0.2	0.82	1380
Matching Layer 1	2220	8357	18.55	0.39	—	—
Matching Layer 2	1996	7792	15.55	0.067	—	—
Matching Layer 3	1797	7233	13	0.068	—	—
Matching Layer 4	1625	6608	10.74	0.067	—	—
Matching Layer 5	1480	5904	8.74	0.068	—	—
Matching Layer 6	1362	5132	6.99	0.067	—	—
Matching Layer 7	1268	4314	5.47	0.068	—	—
Matching Layer 8	1203	3517	4.23	0.067	—	—
Matching Layer 9	1164	2879	3.35	0.068	—	—
Matching Layer 10	1150	2621	3.01	0.067	—	—
Backing Layer	3900	2000	7.8	20	—	—

Supplementary table S1. Simulation parameters for the PMN-PT single crystal transducer with the silicon epoxygradientmetamaterial matching layer. The PMN-PT single crystal has a much better piezoelectric properties and relatively high acoustic impedance[2], Simulation of the PMN-PT single crystal metamaterial matching layer transducer is also carried out by Piezocad. The gradient silicon epoxy metamaterial matching layer was discretized into 10 sub-layers, and the simulationacoustic parameters of each sub-layer was presented in this table.

Supplementary Notes

Supplementary Note 1. Measurement of the equivalent acoustic parameters of 1-3 composite

After extracting the metamaterial matching layer as showed in Figure 1, the remaining 1-3 type silica-epoxy composite was prepared to test the acoustic impedance. Equivalent density of this composite was measured to be 1950 kg m^{-3} by the Archimedes principle and its normal longitudinal velocity was 5837 m s^{-1} which was obtained by the inserting substitution method underwater[2], then its specific acoustic impedance was calculated to be 11.4 MRayls. For the pure Epoxy resin, its acoustic impedance was measured to be 3.0 MRayls with the density of 1150 kg m^{-3} and longitudinal velocity 2650 m s^{-1} through the same method. So the impedance changing range of the matching layer was estimated to be between 3.0 MRayls and 11.4 MRayls this agrees with the theoretical calculation well.

Supplementary Note 2. Theoretical calculation of the sound transmittance through this metamaterial matching layer

For a material whose density and bulk modulus is uniformly distributed in space, the one-dimensional plane monochromatic wave equation has the form below,

$$\frac{\partial^2}{\partial x^2}p(x)+\frac{\omega^2\rho(x)}{\kappa(x)}p(x)-(\frac{\partial\ln\rho}{\partial x})\frac{\partial}{\partial x}p(x)=0 \qquad (1)$$

Here κ(x) and ρ(x) is the space variable, x is the propagation direction. So the wave transmission is dominated not only by bulk modulus κ and density ρ but also by the distribution of these two parameters. Suppose that the exit medium has an infinite length and the backward wave will not reflected,sound intensity transmission coefficient can be expressed by the material impedance of the incident medium and the acoustic impedance of the interface by the following formula:

$$t_I=1-\frac{\left|Z|_{x=0}-Z_0\right|}{\left|Z|_{x=0}+Z_0\right|} \qquad (2)$$

Here Z represents the interface impedance and Z_0 represent the incident material impedance. The conventional problems with homogeneous interlayer (thickness d) can be solved by transferring the impedance of the exit medium through such formula:

$$Z_s\big|_{x=0}=R\frac{Z_s\big|_{x=d}+jR\tan kd}{R+jZ_s\big|_{x=d}\tan kd} \qquad (3)$$

However, this formula is invalid for the complex medium which has a distribution of bulk modulus and density. For this reason, an impedance transfer equation substituting the impedance transfer formula is given by which the impedance of the interface can be calculated.

On the other hand, when two expressions $Z(x)=\frac{p(x)}{v(x)}$ and $\Phi=\frac{p'}{p}=-\frac{j\omega\rho}{Z}$ were substituted back into equation 1, a more concise equation can be obtained as showed below. $\frac{p''(x)}{p(x)}+\omega^2\frac{\rho(x)}{\kappa(x)}+\alpha\Phi=0$. Moreover, by simplifying this equation with $\Phi'=\frac{p''}{p}-\Phi^2$, the impedance transfer equation can then be derived $\Phi'+\omega^2\frac{\rho(x)}{\kappa(x)}+\alpha\Phi+\Phi^2=0$, or the full expression form:

$$-j\omega\left(\frac{\rho(x)}{Z}\right)' - (\frac{\partial \ln \rho}{\partial x})\frac{j\omega\rho(x)}{Z} + \frac{\omega^2\rho(x)}{\kappa(x)} - \left(\frac{\omega\rho(x)}{Z}\right)^2 = 0 \quad (4)$$

Noted that, this is a differential equation of first order which means it can be solved by only one boundary condition. In other words,the impedance of the incident boundary can be calculated by the impedance of the exit boundary which is equal toρc (according to the density and sound velocity of the exit propagation material).

And for elastic wave propagate in solid material, when the longitudinal mode is only considered, by analogy with the compressional wave in the fluid, the bulk modulus κ(x) could be replaced by the equivalent elastic matrix coefficient[1]

$$\overline{C}_{33} = n[c_{11}^{'} - \frac{2(1-n)(c_{12}^{'} - c_{12})^2}{n(c_{11} + c_{12}) + (1-n)(c_{11}^{'} + c_{12}^{'})}] + (1-n)c_{11} \quad (5)$$

In the case sound wave travel from the piezoelectric ceramic to the epoxy resin through this matching material, it is convenient to solve this impedance transfer equation by the computer numerical calculation software Mathematica, and then the calculated transmittance is derived that is showed in Fig. 3b.

Supplementary References

1. Smith, W.A. & Auld, B.A. Modeling 1-3 composite piezoelectrics: thickness-mode oscillations. *IEEE Trans. Ultra. Ferro. Freq. Contr.* **38,** 40-47 (1991).
2. Li, H., Li, Y.C., Zhou, D., Peng, J., Luo, H.S. & Dai, J.Y. Application of PMN-PT single crystal in a 3.2 MHz phased-array ultrasonic medical imaging transducer. *Proc. IEEE. Ultrason. Symp.***1.** 569-571 (2007).
3. Grewe, M. G., Gururaja, T. A., Shrout, T. R. & Newnham, R. E. Acoustic properties of particle/polymer composites for ultrasonic transducer backing applications. *IEEE Trans. Ultra. Ferro. Freq. Contr.* **37,** 506-514 (1990).
